# Pretemporal anteromedial interdural approach to Meckel’s cave: How I do it

**DOI:** 10.1007/s00701-026-06802-w

**Published:** 2026-02-20

**Authors:** Sabino Luzzi, Matìas Baldoncini, Gianluca Lorenzo Fabozzi, Tevfik Serhan Bora, Juan Villalonga, Alvaro Campero

**Affiliations:** 1 Department of Medicine, Surgery, and Pharmacy, University of Sassari, Via Roma 151, 07100 Sassari, Sardinia Italy; 2 Department of Neurosurgery, AOU Sassari, Azienda Ospedaliera Universitaria, Ospedale Civile SS. Annunziata, Sassari, Sardinia Italy; 3 Laboratory of Microsurgical Neuroanatomy, Second Chair of Gross Anatomy, School of Medicine, University of Buenos Aires, Buenos Aires, Argentina; 4 Department of Neurological Surgery, Hospital San Fernando, Buenos Aires, Argentina; 5 Department of Neurological Surgery, Padilla Hospital, Tucumán, Argentina

**Keywords:** Interdural approach, Meckel’s cave, Pretemporal anteromedial corridor, Skull base surgery, Trigeminal schwannoma

## Abstract

**Background:**

The pretemporal anteromedial interdural approach provides direct extradural access to Meckel’s cave by exploiting the natural plane between the temporal dura propria and the meningeal layer of the lateral wall of the cavernous sinus. After a pretemporal craniotomy, the foramen rotundum guides interdural dissection along V2 toward the corridor between V1 and V2, enabling controlled exposure of the Gasserian ganglion and proximal trigeminal rootlets. Tumor resection proceeds through internal debulking and circumferential dissection, with extension into the posterior fossa when necessary.

**Methods:**

In this *How I Do It* article, we present the key technical steps of the pretemporal anteromedial interdural approach to Meckel’s cave, emphasizing anatomical landmarks and operative nuances.

**Conclusion:**

This approach allows safe and effective removal of trigeminal schwannomas while limiting cavernous sinus manipulation and reducing morbidity.

**Supplementary Information:**

The online version contains supplementary material available at 10.1007/s00701-026-06802-w.

## Introduction

The middle cranial fossa is characterized by a double-layer dural configuration that enables interdural dissection along the lateral wall of the cavernous sinus. In the 1990 s, Dolenc popularized the so-called “epidural” approach to lesions of Meckel’s cave and the cavernous sinus [3, 4], later recognized as truly “interdural” because the surgical plane lies between the meningeal and periosteal dural layers [7, 13].

The term *epidural approach* remains however appropriate, as emphasized by Roche and colleagues, since the technique relies on a dissection plane that is entirely extradural [11].

For Meckel’s cave tumors, the interdural approach converts multi-compartmental lesions into a unified parasellar surgical corridor spanning from the superior orbital fissure to the porus trigeminus. The posteromedial portion of Meckel’s cave represents the most challenging area to expose because of the anatomical overlap between the upper half of the Gasserian ganglion and the inferior third of the lateral wall of the cavernous sinus [1, 5, 9, 10, 12]. Three interdural corridors to the posteromedial portion of Meckel’s cave have been described—infratrochlear transcavernous, anteromedial (AM), and anterolateral—each related to a distinct cavernous sinus triangle [10]. The AM corridor, delimited by the first and second divisions of the trigeminal nerve, provides the most straightforward, favorable, and safe route to the posteromedial portion of Meckel’s cave [8].

The present *How I Do It* contribution details the microsurgical anatomy, stepwise exposure, and technical nuances of the pretemporal anteromedial interdural approach to Meckel’s cave.

## Relevant surgical anatomy

**Fig. 1:**
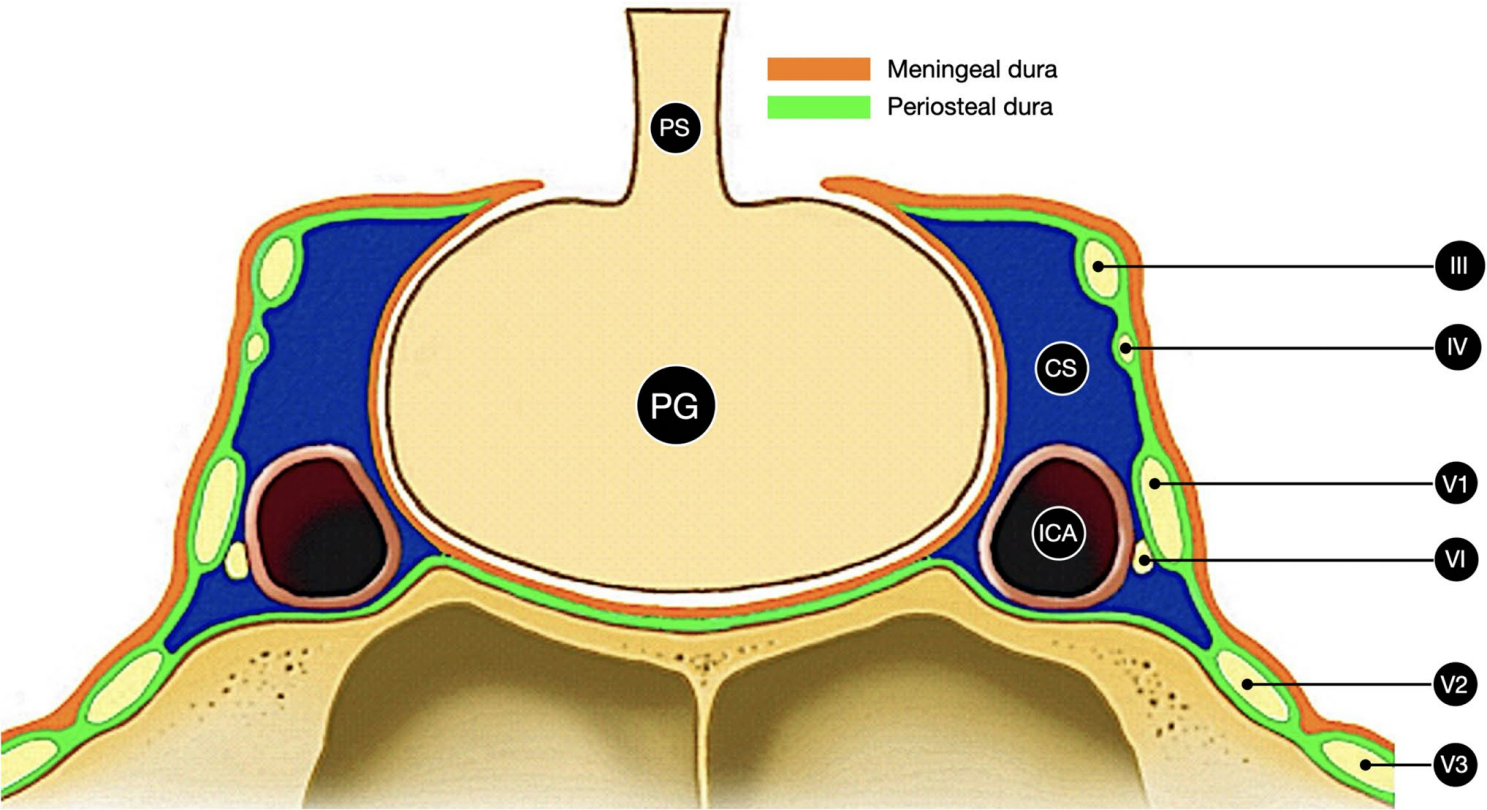
Schematic drawing illustrating the periosteal and meningeal dural layers forming the lateral wall of the cavernous sinus and Meckel’s cave. PS: pituitary stalk. PG: pituitary gland. CS: cavernous sinus. ICA: internal carotid artery. III: third cranial nerve. IV: trochlear nerve. V1: first trigeminal division. V2: second trigeminal division. V3: third trigeminal division. VI: sixth cranial nerve

Meckel’s cave is a truly interdural recess located in the posterolateral portion of the parasellar region, where the surgical plane lies between the meningeal and periosteal dural layers (Fig. 1). It extends between the lateral wall of the cavernous sinus and the petrous apex, enclosing the trigeminal (Gasserian) ganglion and the proximal rootlets of the fifth cranial nerve [10]. These rootlets divide behind the ganglion to form the so-called *pars triangularis*. The nerve then traverses the *porus trigeminus* to reach the pons [6]. The cavity is lined by meningeal dura and contains a thin arachnoid sleeve forming the trigeminal nerve cistern [13]. At the medial-inferior margin of the Meckel’s cave, the petrolingual ligament bridges the petrous apex to the sphenoid lingula, forming the posteroinferior attachment of the cavernous sinus wall and providing a fixed surgical dural landmark [14].

**Fig. 2:**
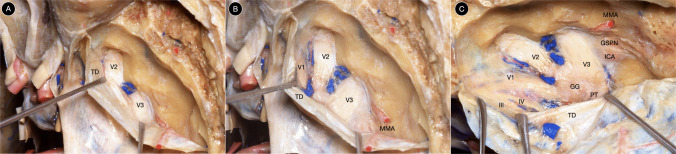
**A-C** Anatomical dissection of the middle fossa illustrating the trigeminal ganglion within Meckel’s cave and the periosteal–meningeal dural configuration of the lateral wall of the cavernous sinus, which forms the basis of the interdural surgical corridor. TD: temporal dura. III: third cranial nerve. IV: trochlear nerve. V1: first trigeminal division. V2: second trigeminal division. V3: third trigeminal division. ICA: internal carotid artery. MMA: middle meningeal artery. GSPN: greater superficial petrosal nerve. GG: Gasserian ganglion. PT: pars triangularis

The parasellar space, of which Meckel’s cave constitutes the posterior compartment, lies lateral to the sella turcica and medial to the temporal dura. It includes the cavernous sinus and the paraclinoid region, bounded superiorly by the meningeal dura and carotid–oculomotor membrane, inferiorly by the periosteal layer of the middle cranial fossa, medially by the pituitary gland and sphenoid sinus, and laterally by the temporal dura and superior orbital fissure. Within this complex dural architecture, the internal carotid artery follows an S-shaped course surrounded by the venous plexus of the cavernous sinus and crossed by cranial nerves III, IV, V1, V2, and VI within distinct dural layers (Fig. 2).

## Description of the technique

### Position

**Fig. 3:**
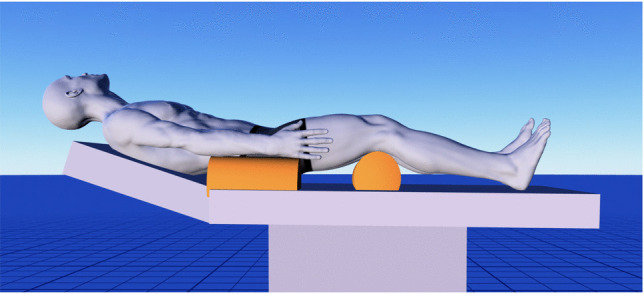
Surgical position

The patient is positioned supine with the head elevated above the level of the heart, rotated 30° to the contralateral side, and flexed 20° to align the sagittal plane parallel to the floor. In this position, the zygomatic arch represents the highest point in the surgical field (Fig. 3).

### Craniotomy

**Fig. 4:**
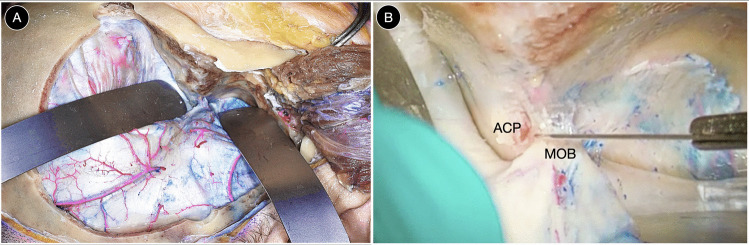
**A** Pterional pretemporal craniotomy (**B**) Division of the meningo-orbital band. This maneuver allows progressive interdural peeling of the lateral wall of the cavernous sinus and Meckel’s cave

A standard pterional pretemporal craniotomy with a low temporal cut flush to the middle fossa floor is performed. The lateral sphenoid ridge is flattened, and the temporal base drilled to achieve a horizontal trajectory over the middle fossa (Fig. 4A).

### Pretemporal anteromedial interdural approach

The pretemporal anteromedial interdural approach begins with identification and sectioning of the meningo-orbital band, which exposes the plane between the temporal dura propria and the meningeal dural layer of the lateral wall of the cavernous sinus (Fig. 4B). This maneuver provides access to the true interdural space. The foramen rotundum serves as the key starting landmark, guiding the dissection along the proximal segment of the maxillary nerve (V2). The interdural dissection is then extended posteriorly and medially along V2 toward the ophthalmic division (V1), progressively widening the surgical corridor. The space between V1 and V2 defines the pretemporal anteromedial corridor, which provides direct access to the posteromedial portion of Meckel’s cave, limited posteriorly by the porus trigeminus. The meningeal dural envelope of Meckel’s cave is incised longitudinally to expose the Gasserian ganglion and the proximal trigeminal rootlets. After internal tumor debulking, circumferential capsular dissection is performed under direct visualization, preserving the integrity of the trigeminal divisions. In dumbbell-shaped lesions, the expanded Meckel’s cave allows controlled extension into the posterior fossa through the enlarged porus trigeminus. Reconstruction is completed by re-approximating the meningeal dural layers, replacing the bone flap, and ensuring a watertight closure.

### Surgical steps for pretemporal anteromedial interdural approach

1. Meningo-orbital band management: The meningo-orbital band is identified and cut to expose the plane between the temporal dura propria and the meningeal dural layer of the lateral wall of the cavernous sinus, providing the true interdural entry point.
2. Starting landmark: The foramen rotundum serves as the key landmark to initiate interdural dissection and identify the proximal segment of the maxillary nerve (V2).
3. Interdural dissection: The dissection proceeds posteriorly and medially along V2 within the interdural space, extending toward the ophthalmic division (V1).
4. Pretemporal anteromedial corridor identification: The space between V1 and V2 is recognized as the anteromedial (AM) interdural corridor leading to the posteromedial portion of Meckel’s cave, whose posterior limit is defined by the porus trigeminus.
5. Opening of Meckel’s cave: The meningeal dural envelope is incised longitudinally to reach the Gasserian ganglion and proximal trigeminal rootlets.
6. Tumor debulking and dissection: Internal decompression and circumferential capsular dissection are performed under direct visualization, preserving V1–V3 integrity.
7. Posteromedial extension: In dumbbell-shaped lesions, the expanded Meckel’s cave allows controlled progression into the posterior fossa through the enlarged porus trigeminus.
8. Reconstruction: The meningeal dural layers are re-approximated and the bone flap replaced.

## Indications

The pretemporal anteromedial interdural approach is indicated for trigeminal schwannomas centered in Meckel’s cave and in those 'dumbbell' with limited posterior fossa extension through the porus trigeminus. It allows one-stage exposure of the Gasserian ganglion and posteromedial portion of Meckel’s cave without entering the cavernous sinus [2, 8, 13].

**Fig. 5:**
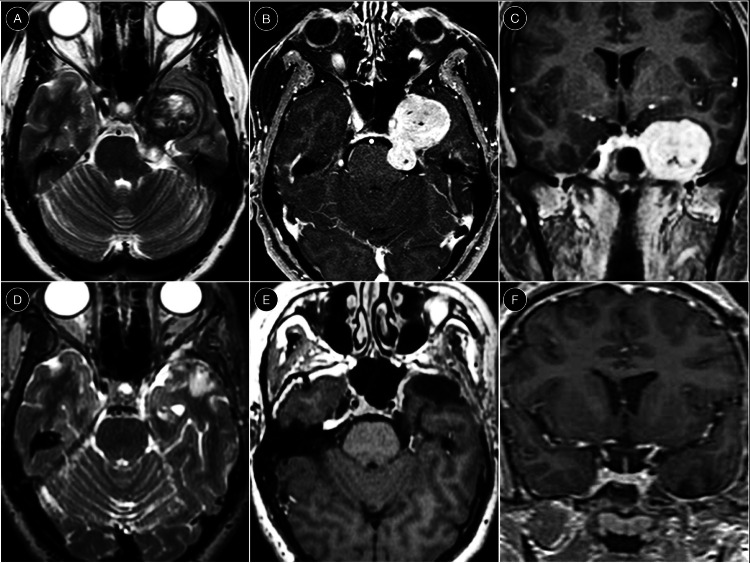
**A-C** Preoperative axial T2-weighted MRI (**A**), and axial (**B**) and coronal (**C**) T1-weighted gadolinium-enhanced MRI. **D-F** Postoperative axial T2-weighted (**D**) and axial (**E**) and coronal (**F**) T1-weighted gadolinium-enhanced MRI demonstrating the extent of resection

**Fig. 6:**
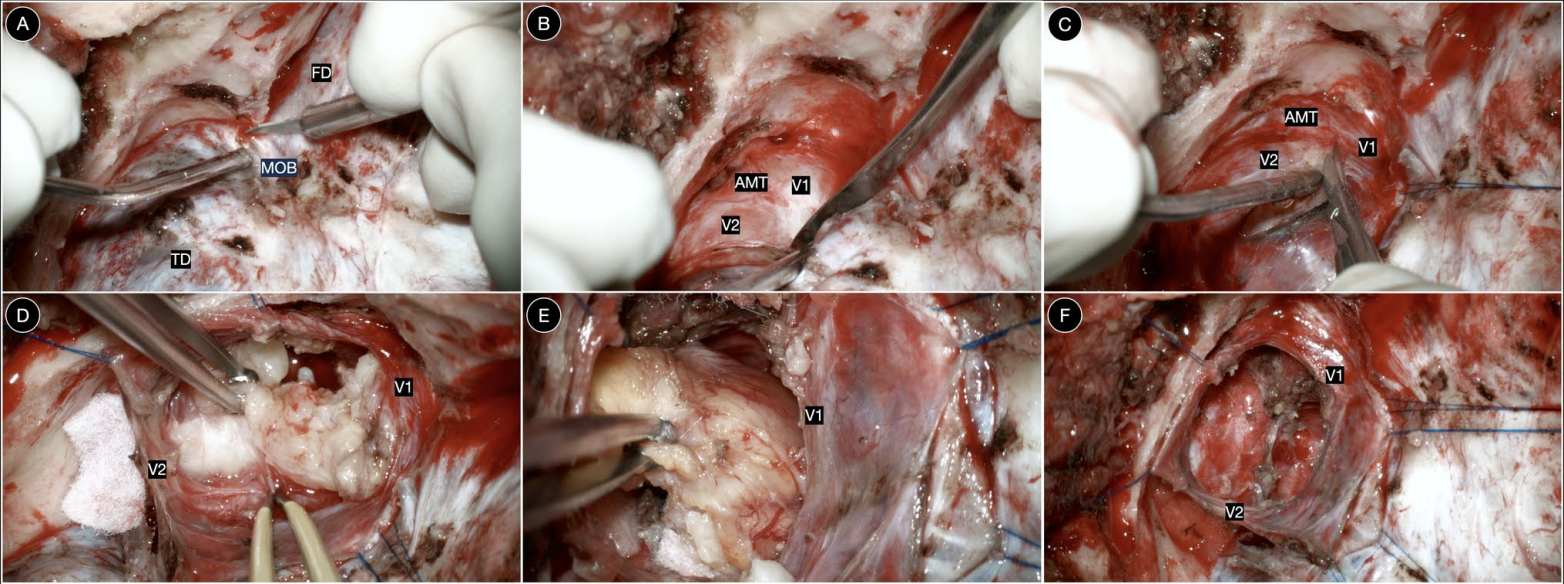
Intraoperative sequence of the illustrative case illustrating the pretemporal anteromedial interdural approach to Meckel’s cave and stepwise tumor debulking and resection (**A-F**)

In the herein reported illustrative case, a 62-year-old female with long-standing left facial pain refractory to medical therapy was diagnosed with a left Jefferson type C trigeminal schwannoma, with a small posterior fossa component (Fig. 5A-C). A pretemporal anteromedial interdural approach was performed, using exclusively the anteromedial corridor to achieve gross-total resection of the middle fossa portion through the expanded Meckel’s cave. The posterior fossa component was removed via the enlarged porus trigeminus facilitated by tumor-induced bone scalloping of the suprameatal area (Fig. 6). Postoperative MRI confirmed gross-total resection (Fig. 5D-F). The patient was discharged on postoperative day 3 without neurological deficits.

The key surgical steps of pretemporal anteromedial interdural approach to Meckel’s cave are demonstrated in Video 1.

## Limitations

The pretemporal anteromedial interdural approach cannot be substantially expanded without additional bone removal such as anterior clinoidectomy or anterior petrosectomy. Its tangential trajectory restricts access to large or deeply posterior extensions not widening the porus trigeminus. Tumor-induced distortion of the trigeminal divisions may complicate anatomical orientation, and inadvertent entry into the cavernous sinus carries a risk of venous bleeding and cranial nerve injury. When a clear dissection plane between the tumor and the trigeminal fibers is difficult or absent, this corridor may become significantly limited in terms of surgical freedom.

## How to avoid complications

- Remain within the interdural plane to avoid entering the cavernous sinus and causing venous bleeding or nerve injury.
- Limit peeling around V2 and V1 to prevent postoperative hypoesthesia.
- When opening the porus trigeminus, coagulate and cut the superior petrosal sinus under direct vision to prevent avulsion hemorrhage.
- Preserve the oculomotor, trochlear, and abducens nerves during the peeling.
- In cases where a clear dissection plane between the tumor and the trigeminal fibers is difficult or impossible to obtain, a subtotal rather than radical resection should be considered as the safer and more valuable option, given the risk of postoperative permanent hypesthesia [2].

## Specific information to patient

The pretemporal anteromedial interdural approach is more anatomical and conservative than alternative routes to Meckel’s cave, as it preserves the integrity of the cavernous sinus and minimizes brain retraction. In patients with trigeminal schwannomas centered in Meckel’s cave or presenting as dumbbell lesions with limited posterior fossa extension, this approach allows complete tumor removal through a single procedure, avoiding the need for combined or staged surgeries. However, patients should be informed of potential risks such as transient or permanent facial hypoesthesia, diplopia, or other cranial nerve deficits, as well as the rare possibility of cerebrospinal fluid leakage or venous bleeding from the cavernous sinus.

## Conclusion

The pretemporal anteromedial interdural approach provides a direct, extradural, and anatomically guided route to Meckel’s cave. By exploiting the natural interdural plane and the anteromedial corridor between V1 and V2, it enables safe and effective resection of trigeminal schwannomas centered in Meckel’s cave or with limited posterior fossa extension, while preserving the cavernous sinus and minimizing brain manipulation. This approach represents a refined, conservative, and function-preserving option in the modern microsurgical management of Meckel’s cave lesions.

## Supplementary Information

Below is the link to the electronic supplementary material.

ESM 1 Supplementary Material 1 (MOV 1224459 KB)

## Data Availability

No datasets were generated or analysed during the current study.

## Declarations

Informed consent was obtained from the patient reported in the illustrative case.
